# Hinge-initiated Primer-dependent Amplification of Nucleic Acids (HIP) – A New Versatile Isothermal Amplification Method

**DOI:** 10.1038/s41598-017-08067-x

**Published:** 2017-08-09

**Authors:** Jens Fischbach, Marcus Frohme, Jörn Glökler

**Affiliations:** Division of Molecular Biotechnology and Functional Genomics, Technical University of Applied Sciences Wildau, Hochschulring 1, Wildau, 15745 Germany

## Abstract

The growing demand for cost-effective nucleic acid detection assays leads to an increasing number of different isothermal amplification reaction methods. However, all of the most efficient methods suffer from highly complex assay conditions due to the use of complicated primer sets and/or auxiliary enzymes. The present study describes the application of a new linker moiety that can be incorporated between a primer and a secondary target binding site which can act both as a block to polymerase extension as well as a hinge for refolding. This novel “hinge-primer” approach results in an efficient regeneration of the primer binding site and thus improves the strand-displacement and amplification process under isothermal conditions. Our investigations revealed that the reaction with forward and reverse hinge-primer including an abasic site is very efficient. The assay complexity can be reduced by combining the hinge-primer with a corresponding linear primer. Furthermore, the reaction speed can be increased by reducing the length of the amplified target sequence. We tested the sensitivity down to 10^4^ copies and found a linear correlation between reaction time and input copy number. Our approach overcomes the usually cumbersome primer-design and extends the range of isothermal amplification methods using a polymerase with strand-displacement activity.

## Introduction

Sensitive and specific detection of nucleic acids is a substantial requirement for clinical diagnosis, forensics, agriculture and environmental sciences as well as in basic and applied molecular research. Among these fields of application the amplification of DNA and/or RNA is the preferred method for analysis^1, 2^. The polymerase chain reaction (PCR) and its further developments are crucial tools that have been applied extensively in molecular diagnostics. PCR represents a primer-dependent target amplification and is based on a cyclic protocol comprising of denaturation of double-stranded DNA, hybridisation of a primer and finally amplification of the template nucleic acid, whereas each step is conducted at different temperatures. Although PCR as the standard method is highly reproducible and sensitive in routine applications, it suffers from limitations such as expensive and complex equipment as well as a higher sensitivity to inhibitory substances in crude samples. The latter limitation has been partially lifted by introduction of new polymerases that are more tolerant to inhibitors such as heparin or humic acids. Nevertheless, PCR is not readily applicable to many field applications which require low-resource settings^3–6^. In recent years, portable PCR-technologies with spatial- or time-dependent temperature cycling have been investigated^7, 8^. To overcome some of the technical and biological disadvantages of PCR, various new amplification reactions have been established as well. Unlike PCR, many alternative methods are conducted at a uniform temperature, hence the reactions can be set up easily and analyzed more rapidly. Such isothermal amplification methods are receiving rising attention due to their speed and cost effectiveness^9–12^. Of all published isothermal methods, the most important are the loop-mediated isothermal amplification (LAMP^13^), strand displacement amplification (SDA^14^), recombinase polymerase amplification (RPA^15^), the helicase dependent amplification (HDA^16^) and nucleic acid sequence based amplification (NASBA^17^). In contrast to thermal denaturation, an efficient strand-separation at constant temperature can be achieved by combining polymerases with strand displacement activity and primers that may fold into functional secondary structures when hybridized to the template DNA. With regard to the reaction mechanism and the number of used components, the above-mentioned methods differ strongly in their complexity. They either suffer from a high number of different primers or from additional enzymes with very different activities. Reactions with many different primer oligonucleotides are prone to form artifacts which may lead to false positive reactions. In order to prevent such artifacts meticulous design is necessary to exclude primer interactions. In addition, primers for methods that rely on the formation of complicated secondary structures such as loops cannot be entirely predicted by software alone, but have to be tested empirically to identify a working set. Other methods may use additional enzymes to the DNA polymerase such as nicking enzymes, recombinases, helicases, single strand DNA binding protein, and auxiliary polymerases. However, as all of these have nucleic acids as a common substrate they compete with each other and slow down the kinetics of the amplification reaction itself. Even more problematic is the provision of an optimal common buffer and thermal conditions in which all enzymes can function synergistically as needed for more complex methods such as NASBA. Generally, LAMP can be described as a primer-intensive method and requires an elaborate primer design. RPA, SDA, NASBA and HDA are more enzyme-intensive reactions and therefore increase the total costs of an assay. LAMP uses primers that generate regions which can snap back at the ends to form loop structures. However, the refolding kinetics are slow and limit the efficiency of the amplification reaction. We sought to develop a new way to improve this refolding step in order to provide a more efficient priming mechanism in an isothermal reaction. Our new isothermal approach combines a simplified reaction set-up by using only two or three unstructured primers and one polymerase with strong strand displacement activity. The present study describes the application of a linker moiety that can be incorporated between a primer and target binding site of a specific oligonucleotide which can act both as a block to polymerase extension as well as a hinge for refolding. Our modified primer facilitates refolding and liberation of the primer binding site for a new primer hybridisation event. This novel “hinge-primer” approach results in an efficient regeneration of the free primer binding site and thus improves strand displacement and amplification process under isothermal conditions.

## Results

**Figure 1:**
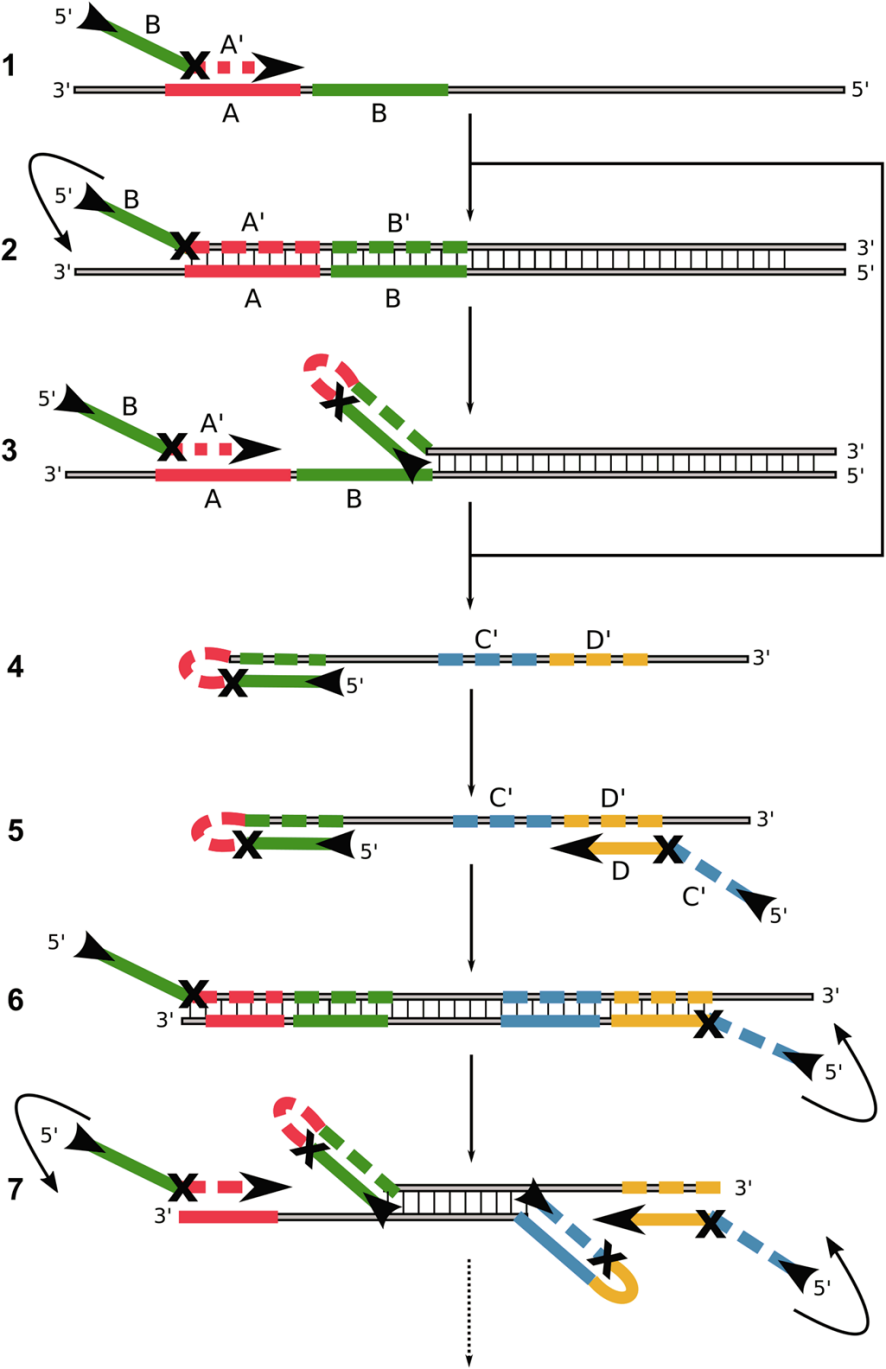
Mechanism of the symmetric Hinge-initiated primer-dependent replication with two hinge-primer. The first stage (A) starts with initial annealing (**1**) of the forward hinge-primer, followed by extension to double strand DNA by *Bst* DNA polymerase (**2**) and refolding to the thermodynamically more stable hairpin structure which liberates (**3**) the initial priming site. These steps are repeated following the release of the sense DNA strand (**4**). The second stage (B) starts with annealing of the reverse hinge-primer to the newly generated and released single strand (**5**) followed by the extension and refolding to a second hairpin structure at both ends (**6**). The initial priming sites on the sense and anti-sense strands are thus liberated by refolding (**7**). Due to continuous recycling of hinge-primer binding sites, the reaction finally results in DNA product accumulation for specific detection. The “X” in the primer represents a blocking modification for the Bst DNA polymerase. Examples are a dSpacer (abasic furan) or an C12-Spacer (hexaethylenglycol). Optionally, an additional outer primer can be applied to increase the speed of initial single strand template generation.

The efficiency of the hinge-initiated isothermal reaction depends on the successive regeneration of the single stranded primer binding sites. The symmetric and cyclic mechanism consists of annealing, extension, displacement and refolding steps (Fig. 1). The amplification reaction is initiated with a sequence-specific oligonucleotide (hinge-fwd) comprising a priming-site (A’) and a secondary binding site (B) that can anneal to the sequence downstream of the priming site (1). Following extension by the DNA polymerase (2), a hairpin end is generated which has a higher melting temperature than the primer binding site alone (3). The secondary binding site is linked to the primer by a base or linker-moiety that essentially blocks the DNA polymerase from copying the secondary binding site. Thus, the secondary binding site remains free to hybridize to the extended sequence for hairpin formation. The primer binding site is liberated for another annealing and extension step while the template strand can repeat all of the previous steps (4). The displaced strand with hairpin structure can now facilitate an additional annealing event by a reverse hinge-primer with he same functionality as the forward hinge-primer (5). After extension by DNA polymerase (6), the secondary binding sites of the forward and reverse hinge-primers are again free to refold to hairpin structures (7). Due to the liberated priming sites on both ends, new priming events can occur. The amplification steps are repeated until primers and nucleotides are exhausted or inhibition occurs by end product accumulation. Due to our previous publication on the real-time and endpoint detection of phytopathogenic microorganisms by LAMP^18^, we started out with the *potato spindle tuber viroid* (PSTVd) as target for our preliminary experiments. Further investigations were performed using the *Escherichia coli* beta galactosidase region (beta-gal) that is conserved in most enterobacteria^19–21^ for confirmation of portability of the hinge amplification principle and optimization.

**Figure 2:**
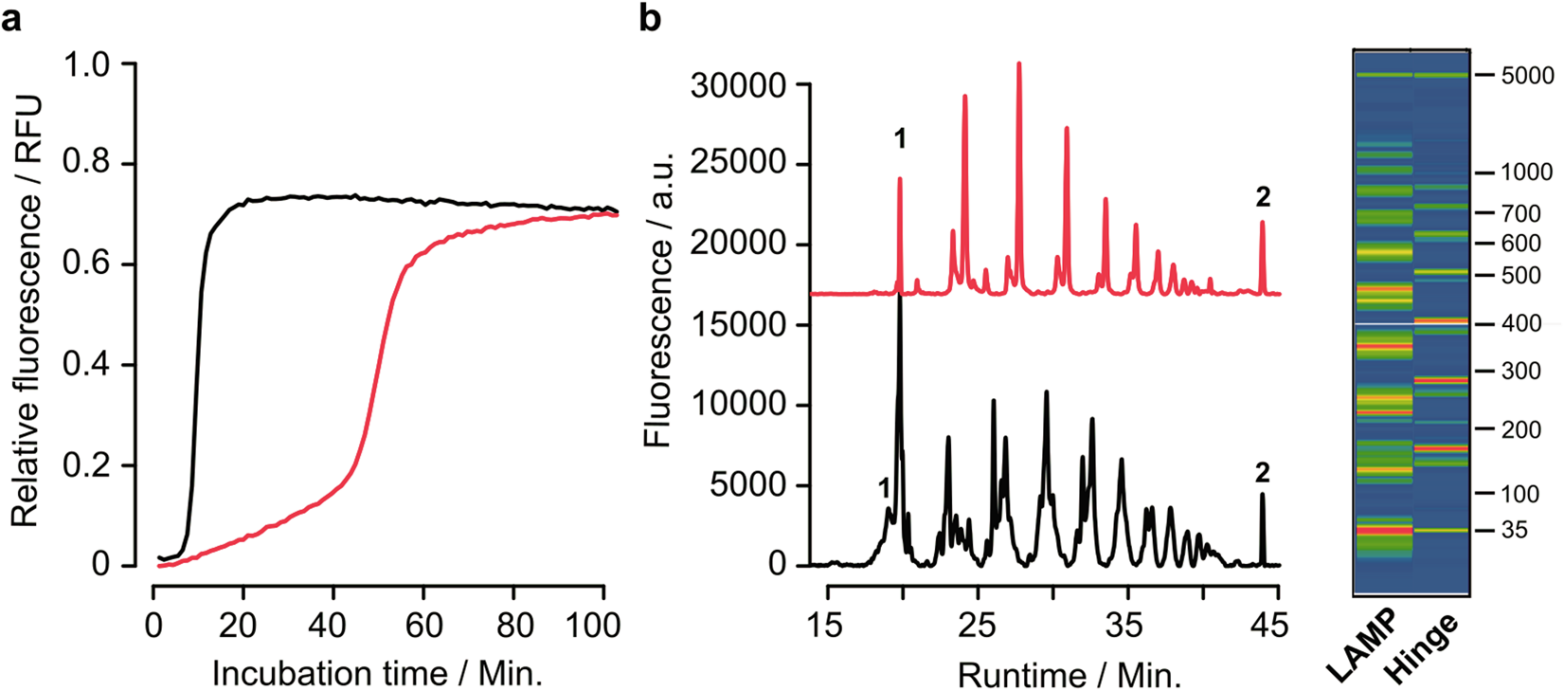
Comparison of the LAMP- and hinge-amplification reaction for the detection of PSTVd. Real-time amplification curves of the LAMP assay (black) and the Hinge-assay (red) at 65 °C for 100 minutes. Fluorescence values are normalized (**a**). Capillary electrophoresis of LAMP (black) and Hinge samples (red) is shown as electropherogram with fluorescence values vs. runtime. Peaks 1 and 2 represent the internal size-standard (35 bp and 5000 bp). The virtual gel image on the right depicts the amplified DNA products of both samples (**b**). The size marker ranges from 35 bp to 5000 bp. Both reactions contained 0.2 ng of total input PSTVd DNA.

**Figure 3:**
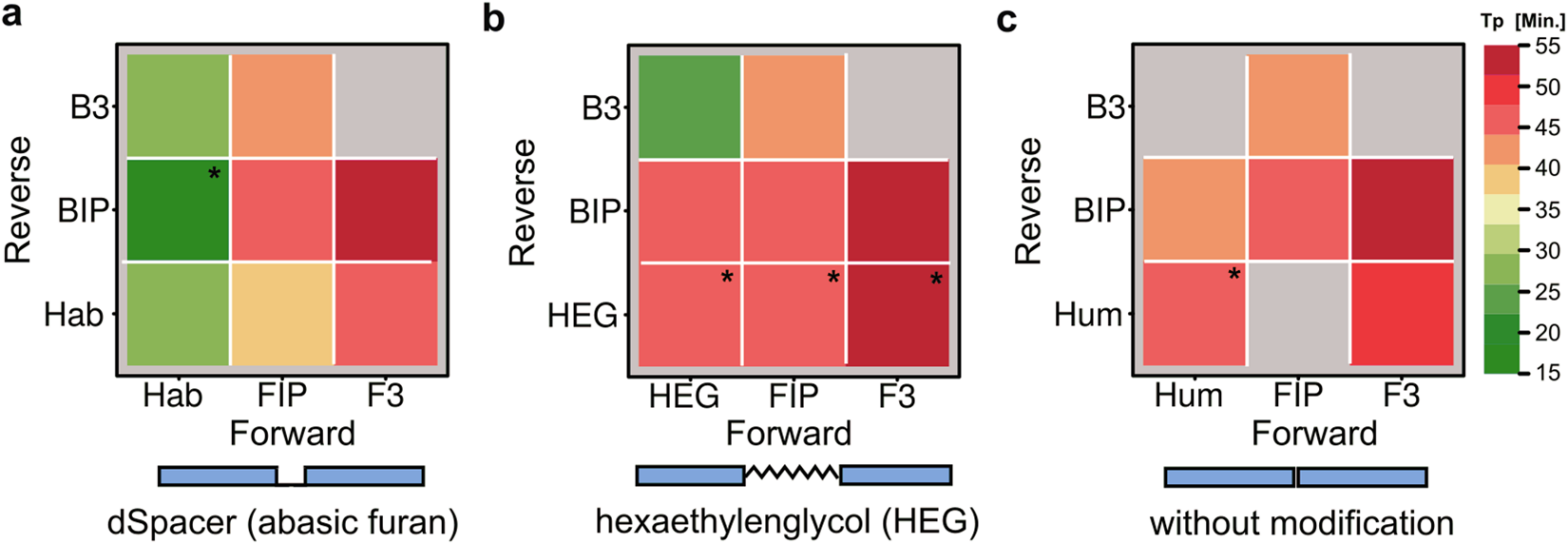
Heatmap representation of the reaction kinetics dependent on primer-modifications. The colors represent different time-to-positivity (Tp) values for each primer combination. (**a**) Hinge-primer reactions with dSpacer (Hab), (**b**) hinge-primer with C12-Spacer (HEG), (**c**) without any modification (Hum). Thus green represents fast and red slow amplification kinetics. B3 and F3 are PCR-like primers used in LAMP, BIP and FIP are loop-forming LAMP primers. The asterix (*) represents control reactions without template DNA that showed a detectable amplification signal after 45 minutes. Grey fields show reactions without detectable amplification signal.

The sequences of the oligonucleotides that were used for both targets are listed in Table 1. The hinge-amplification depends on a modification to prevent extension beyond the secondary binding site. It is known that non-ribose linkers such as hexaethyleneglycol efficiently block a polymerase extension in an oligonucleotide. Some polymerases such as the archaeal family B polymerases are also known to be inhibited by deaminated bases such as uracil or hypoxanthine^22^. However, abasic sites represent efficient roadblocks to most polymerases as well. The so-called dSpacer is provided as a simple phosphoramidite mimicking an abasic site that can be introduced during conventional oligonucleotide synthesis. To test the general amplification efficiency under typical isothermal reaction conditions we used hinge-oligonucleotides (Table 1) with a dSpacer modification and published LAMP oligonucleotides that are specific for PSTVd. Both isothermal reactions were carried out with 0.2 ng PSTVd-DNA. The isothermal amplification kinetics of each reaction was compared by observing the amplification curves (Fig. 2a). In comparison to LAMP, the hinge-initiated reaction resulted in a longer initial lag phase before an exponential phase was observed. The LAMP resulted in a strong signal increase after 12 minutes whereas the hinge-reaction with two hinge-primers reached the exponential phase after 45 minutes with the same amount of DNA input. The electropherogram of both reaction products displays a ladder-like DNA structure (Fig. 2b). The peaks in the electropherogram of the LAMP reaction have a higher fluorescence amplitude but are also broader as compared to the hinge reaction. The DNA banding patterns were clearly distinguishable between both reactions. The virtual gel image of both samples indicates different amounts of amplified DNA for both reactions (Fig. 2b right) as well as different product sizes. Due to the lower reaction speed observed in the first experiment, we sought to explore a combination of one hinge-primer with a loop-forming primer (FIP and BIP) of the LAMP reaction to improve the reaction speed. Additionally, we investigated a hinge-primer modification based on a hexaethylenglycol-spacer (HEG). A hinge-primer without any modification was used as a control. To test whether one hinge-primer might be sufficient for amplification we also explored a combination of hinge-reaction with conventional linear primers. Therefore, we checked all possible combinations of hinge primers including the dSpacer, HEG or without modification with the corresponding reverse primer as either standard PCR primer or the loop-forming primer of the LAMP format. The resulting time-to-positivity values (Tp) are illustrated as a heatmap (Fig. 3) and listed in supplementary information (Table S1).

**Table 1:** Overview of LAMP- and Hinge-primer for detection of PSTVd and beta-gal (*E.coli*).

Locus: *Potato Spindle Tuber Viroid* (NC_002030.1)	
LAMP primer	Primer sequence (5′ – 3′)
F3	AAAAAGGACGGTGGGGAG
B3	CCCCGAAGCAAGTAAGATAG
FIP (F1 + F2)	GGAAGGACACCCGAAGAAAGG-GCCGACAGGAGTAATTCC
BIP (B1 + B2)	GCTGTCGCTTCGGCTACTAC-AGAAAAAGCGGTTCTCGG
Lf	GGTGAAAACCCTGTTTCGG
Lr	CGGTGGAAACAACTGAAGC
**Hinge-primer**	
PSTVd-um-HF	GGAAGGACACCCGAAGAAAGG**-**CCGAAACAGGGTTTTCACC
PSTVd-um-HR	GCTGTCGCTTCGGCTAC**-**TTCAGTTGTTTCCACCG
PSTVd-ab-HF	GGAAGGACACCCGAAGAAAGG**[dSpacer]**CCGAAACAGGGTTTTCACC
PSTVd-ab-HR	GCTGTCGCTTCGGCTAC**[dSpacer]**TTCAGTTGTTTCCACCG
PSTVd-HEG-HF	GGAAGGACACCCGAAGAAAGG**[SpacerC12]**CCGAAACAGGGTTTTCACC
PSTVd-HEG-HR	GCTGTCGCTTCGGCTAC**[SpacerC12]**TTCAGTTGTTTCCACCG
**Locus:** ***Escherichia coli*** **str. K12 substr. DH10B (CP000948.1)**	
**Hinge primer**	**Primer sequence (5′ – 3′)**
LZ-HF	CCAGCGCCCGTTG**[dSpacer]**CTCGGCGTTTCATCTGTG
LZ-HR	CGGTGATGGTGCTGC**[dSpacer]**AGATAACTGCCGTCACTCC
LZL-389	ATGAAAGCTGGCTACAGGAAGGCC
LZR-653	GGTTTATGCAGCAACGAGACGTCA
LZF-2	CTCGGCGTTTCATCTGTG
LZR-2	AGATAACTGCCGTCACTCC
LZF-0	TCGGTTACGGCCAGG
LZR-0	AGGCGGTTTTCTCCGG

The bold letters in the sequence denotes the location of the blocking modification (um: unmodified, ab: dSpacer and HEG: SpacerC12). For the detection of PSTVd two modifications and a control were used: dSpacer (abasic furane), c12-Spacer (hexaethyleneglycol) or no modification. For the detection of *E.coli*, only the dSpacer was used. All oligonucleotides were HPLC purified.

The analysis of primer modifications revealed differences in the time-to-positivity (Tp) values^23^ within and between each group (Fig. 3a–c). The first group consists of the hinge-primer with a dSpacer (abasic site) modification as well as the loop-forming primer of the LAMP (FIP and BIP) and the outer primer (F3 and B3). The best Tp value was achieved for the combination of Hab/BIP with 15 to 20 minutes. The combinations of Hab forward and Hab reverse as well as Hab forward and B3 resulted in slightly higher values (25–30 minutes). All other combinations resulted in TP values of 40 to 55 minutes. However, the combination Hab forward and BIP also resulted in false-positive values in the non-template control. The second group includes the HEG-modified hinge primers that were combined with the LAMP primer. The best Tp value was achieved for the combination HEG forward and B3 in 25 minutes. Other combinations resulted in higher values in a range of 35 to 55 minutes. All combinations with the HEG reverse hinge primer resulted in false-positive reactions (Fig. 3b). The group consisting of the hinge primer without any modification resulted in Tp values between 40 and 55 minutes. Three combinations did not yield any amplification at all (Fig. 3c). These results suggest that the combination of a dSpacer-modified hinge-primer and a loop-forming LAMP primer is generally feasible. Additionally, the combination of the hinge primer with a normal PCR primer is feasible as well. To overcome the disadvantages of the initial primer-design for the detection of the highly structured PSTVd sequence and the reaction conditions, we established an assay to detect the beta-gal locus of *E.coli* by using a hinge-primer including the dSpacer-modification with and without additional PCR-like primer.

**Figure 4:**
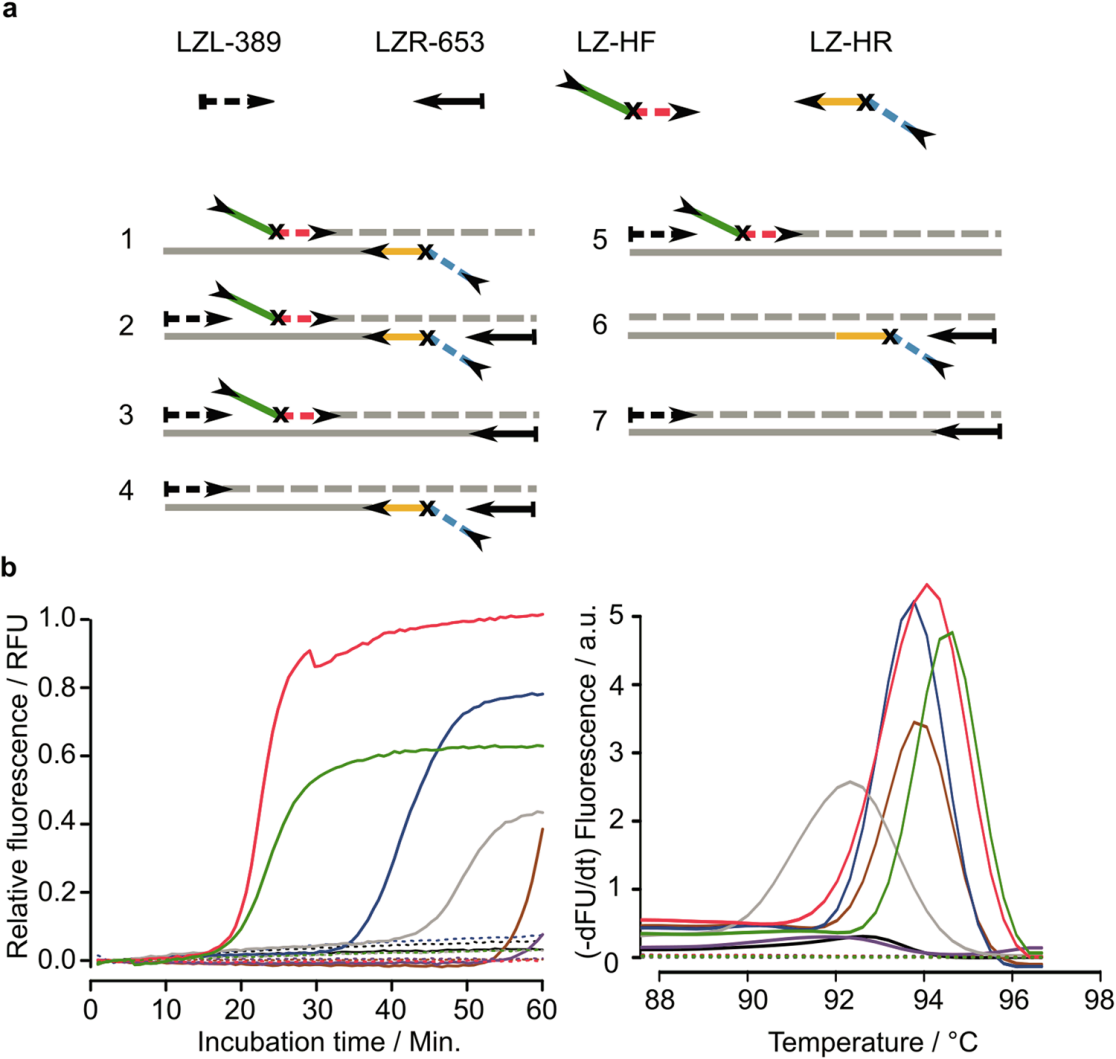
Analysis of the reaction kinetics with respect to different hinge-primer combinations using dSpacer as blocking-site. (**a**) Overview of primer combinations for symmetric reaction (1–2) and asymmetric reaction (3–7) (**b**) Normalised real-time amplification curves for each combination. (**c**) Melting-curve analysis. Red: combination 1, green: combination 2, blue: combination 4, grey: combination 3, brown: combination 6, violet: combination 5 and black: combination 7. Solid lines represent reactions with 5 ng genomic DNA (*E.coli*) and dashed lines are non-template controls.

We tested different primer combinations consisting of the forward and reverse hinge-primer together as well as the forward hinge-primer and reverse hinge-primer alone. The corresponding primer in each combination was a PCR-like primer. Additional outer primer binding to a priming-site upstream to the hinge-primer itself were also investigated (Fig. 4a). The analysis was done by comparing the real-time data of the amplification and a melting curve analysis to confirm specificity (Fig. 4b, right). The comparison of the amplification curves resulted in different endpoint fluorescence signals and Tp values (Fig. 4b, left). The lowest values with 22 minutes were achieved with hinge-primers in separate reactions, independently of additional outer primers. However, both curves could be distinguished according to the reaction kinetics and a higher endpoint signal for the combination without outer primer. The combination of the reverse hinge-primer with the corresponding linear primer and outer primer resulted in a Tp value of 39 minutes. The forward hinge-primer in the same combination resulted in a slightly slower reaction with 47 minutes. The combinations without outer primer yielded the highest Tp values between 50 to 60 minutes. All control reactions without template DNA remained negative. The melting curves revealed only slightly differences with respect to the melting temperature (Fig. 4b). Aside from the sample with the forward hinge-primer, all melting temperatures were between 93 °C and 95 °C. The symmetrical hinge-amplification with both hinge-primer worked directly without any optimization. Further improvement of the amplification reaction has been investigated in subsequent experiments.

Variation of magnesium concentration is known to influence both the reaction kinetics and specificity. Therefore, we tested magnesium chloride concentrations ranging from 4 mM to 10 mM. To compare the data, we calculated the Tp values that are shown in Table 2. The symmetrical hinge-amplification with both hinge primer resulted in Tp values between 17 and 30 minutes. The best values were achieved in the presence of 6 mM magnesium. The Tp values of the forward and reverse hinge-primer alone were determined between 30 to 56 minutes. For both combinations the lowest Tp values was achieved using 6 mM magnesium, which is comparable to the symmetric primer combination.

**Table 2:** Variation of magnesium concentration in the hinge-amplification reaction in combination with three different primers.

Primer combination	Template	Magnesium concentration			
		4 mM	6 mM	8 mM	10 mM
LZ-HF and LZ-HR	+	30 ± 1	17 ± 1	25 ± 1	29 ± 0
	−	NA	46 ± 1	58 ± 1	46 ± 1
LZ-HF and LZR-653	+	56 ± 2	34 ± 1	38 ± 1	45 ± 2
	−	NA	NA	NA	NA
LZL-389 and LZ-HR	+	51 ± 1	39 ± 2	46 ± 1	46 ± 1
	−	NA	NA	NA	NA

The test included both hinge-primer and the forward and reverse primer separately. The magnesium concentration ranges from 4 mM to 10 mM. Time-to-positivity (Tp / minutes) values are listed including standard deviation. NA represents no amplification detected.

Whereas the assay with one hinge primer revealed no false-positive results, the symmetric reaction resulted in a detectable amplification of non-template controls after 45 minutes except for the lowest magnesium concentration. Further experiments were performed with varying primer concentrations (0.8 to 1.4 µM). The data reveals only a slight decrease of the Tp values corresponding with increasing primer concentrations. The symmetric primer combination resulted in false-positive assays for all primer concentrations. However, the Tp values for the single hinge-primer could not be decreased significantly (data not shown).

**Figure 5:**
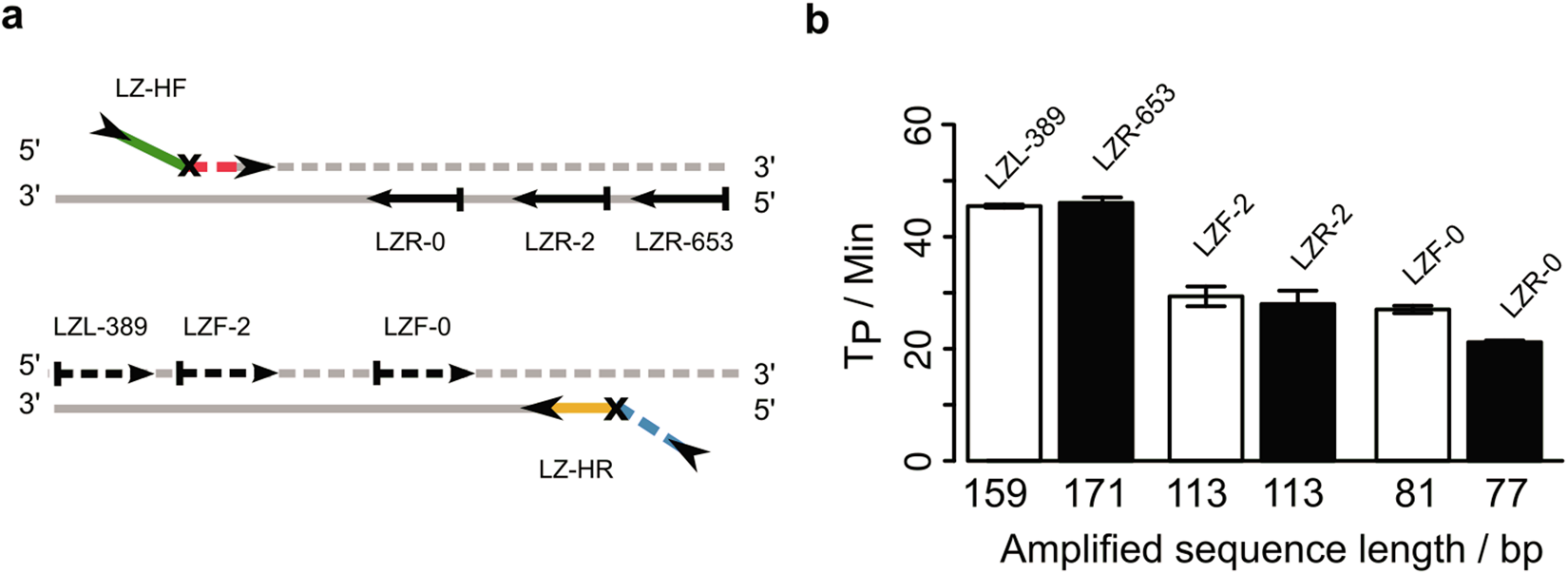
Analysis of the time-to-positivity values of the amplification reactions with decreasing distance of the hinge-primer to the outer primer. (**a**) Binding sites of the primers are illustrated for the Hinge-forward (LZ-HF) and Hinge-reverse (LZ-HR) primer with their respective outer primer LZL-389/LZR-653, LZF/R-2 and LZF/R-0. (**b**) Time-to-positivity values demonstrate the relation of primer distances (length of amplified product) and amplification kinetics. Both orientations of forward hinge-primer (white bars) and the reverse hinge-primer (black bars) are shown. The experiments were carried out in triplicate and standard error bars are shown in the figure.

Another way to increase the reaction speed of the single hinge-primer assays without loss of specificity is shortening of the amplification product. Therefore, we investigated varying distances between primer binding site of each hinge-primer (forward and reverse) and corresponding PCR-like primer. Here we used three different linear primers to achieve distances between 160 and 77 bp of the amplified sequence (Fig. 5a). Time-to-positivity values were outlined as bar chart (Fig. 5b). The Tp values decreased from around 40 to 20 minutes when the distance was shortened down to 77 bp. This decrease could be observed for both hinge-primers.

**Figure 6:**
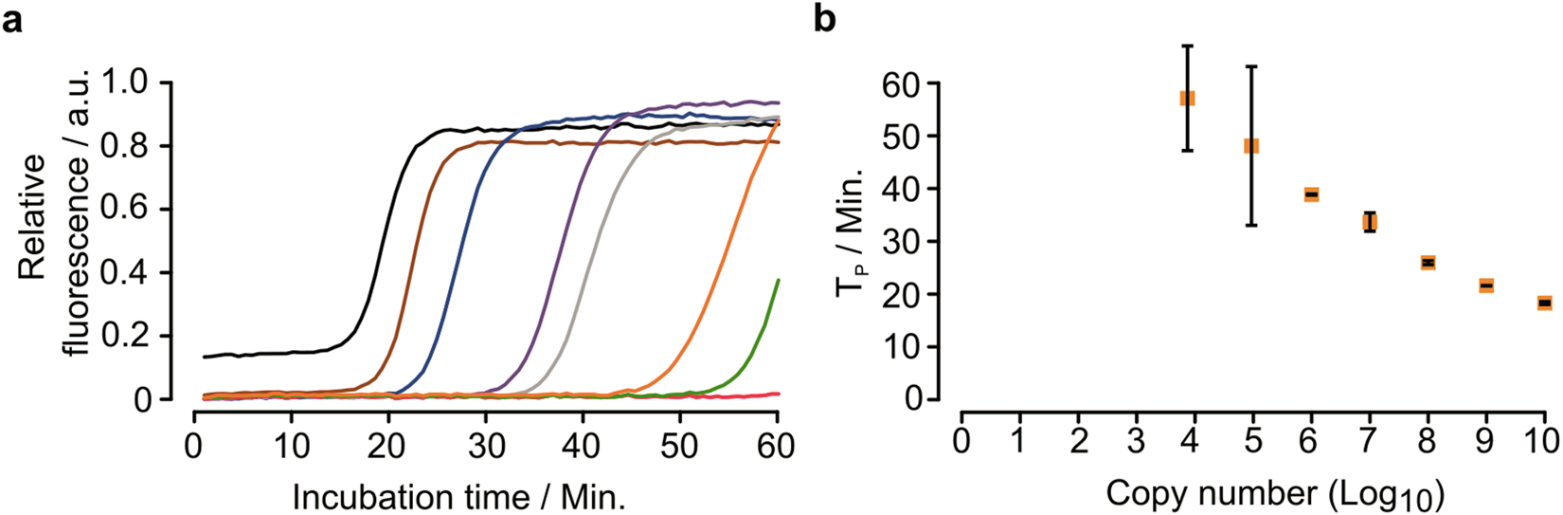
Sensitivity of the HIP-reaction. Detection limit with the LZ-HR primer and the corresponding primer LZF-0 in combination with the primer LZR-653 for the detection of serial diluted genomic *E.coli* DNA for 60 minutes at 65 °C. The time-to-positivity is directly correlated to the input amount of DNA ranging from 10^4^ to 10^10^ copies of the *E.coli* fragment. The coefficient of determination R^2^ = 0.98. The experiments were carried out in triplicate and standard error bars are shown in the figure.

The results indicate that the optimal parameters for an assay using a single reverse hinge-primer in combination with the LZL-0 PCR-like primer are 6 mM magnesium at 65 °C for 60 minutes. To test the performance with complex template of our assay, we finally investigated the detection sensitivity by using serially diluted genomic *E.coli* DNA as template. The calculated input copy number of the genomic DNA ranged from 10^10^ down to 10^4^. The relation between the time-to-positivity and the input amount of DNA is shown in Fig. 6. No Tp values could be calculated for less than 10^4^ copies. The linear range in this assay was between 10^6^ to 10^10^ copies. The coefficient of determination for this range R^2^ = 0.98.

## Discussion

The experiments demonstrate that the new isothermal amplification principle based on the hinge-primers generally works as predicted from theory. The capillary electrophoresis revealed a ladder-like pattern with apparently high molecular-weight DNA products ranging from 100 bp up to more than 1000 bp similar to LAMP. However, due to the overhanging primer ends it is well possible that the products are simply hybridised to form a ladder-like pattern of high molecular weight. In addition, the reaction kinetics differ from LAMP due to a late exponential phase and a slower signal increase. In comparison to LAMP, the overall efficiency of the preliminary hinge-assay for PSTVd with both hinge-primer appears to be lower. In order to improve the reaction different types of linker-structures were compared. Depending on the linker modification, an efficient isothermal amplification with *Bst* DNA polymerase at 60–65 °C is feasible. Ideally, a short C4-spacer should be chosen in order to optimise hairpin primer refolding. The shorter the linker, the more strain is put on the primer binding site which forces the double stranded terminus to open up. Depending on the kind of polymerase, linkers can be made of abasic sites (AP), unlocked nucleic acid (UNA), Uracil, and other potential roadblock modifications. Generally, linkers can also be made of aliphatic building blocks or polyethylenglycol (PEG). The reverse primer can be a standard linear primer. Abasic sites, also called apurinic sites, are one of the most frequent spontaneous lesions in DNA^21^. Incorporated into DNA it creates a strong block to most DNA polymerases. Bst DNA polymerase, which lacks a 3′–5′ exonuclease and proofreading activity is blocked initially at the position immediately 3′ to the abasic site. Both Family A and Family B replicative polymerases have been shown to stall prior to or opposite the abasic site, including T4 DNA polymerase, T7 DNA polymerase, and yeast DNA polymerase δ^24–27^. Abasic sites can be simply generated during oligonucleotide synthesis by incorporation of tetrahydrofuran phosphoramidite (abasic furan). The other tested linker modification is based on hexaethylenglycol (HEG). Both linker-modifications were used as blocking group and hinge element to avoid oligonucleotide extension of the primer extension-site in 3′–5′ direction, thus keeping the secondary binding site single-stranded for an efficient refolding to a hairpin structure.

**Figure 7:**
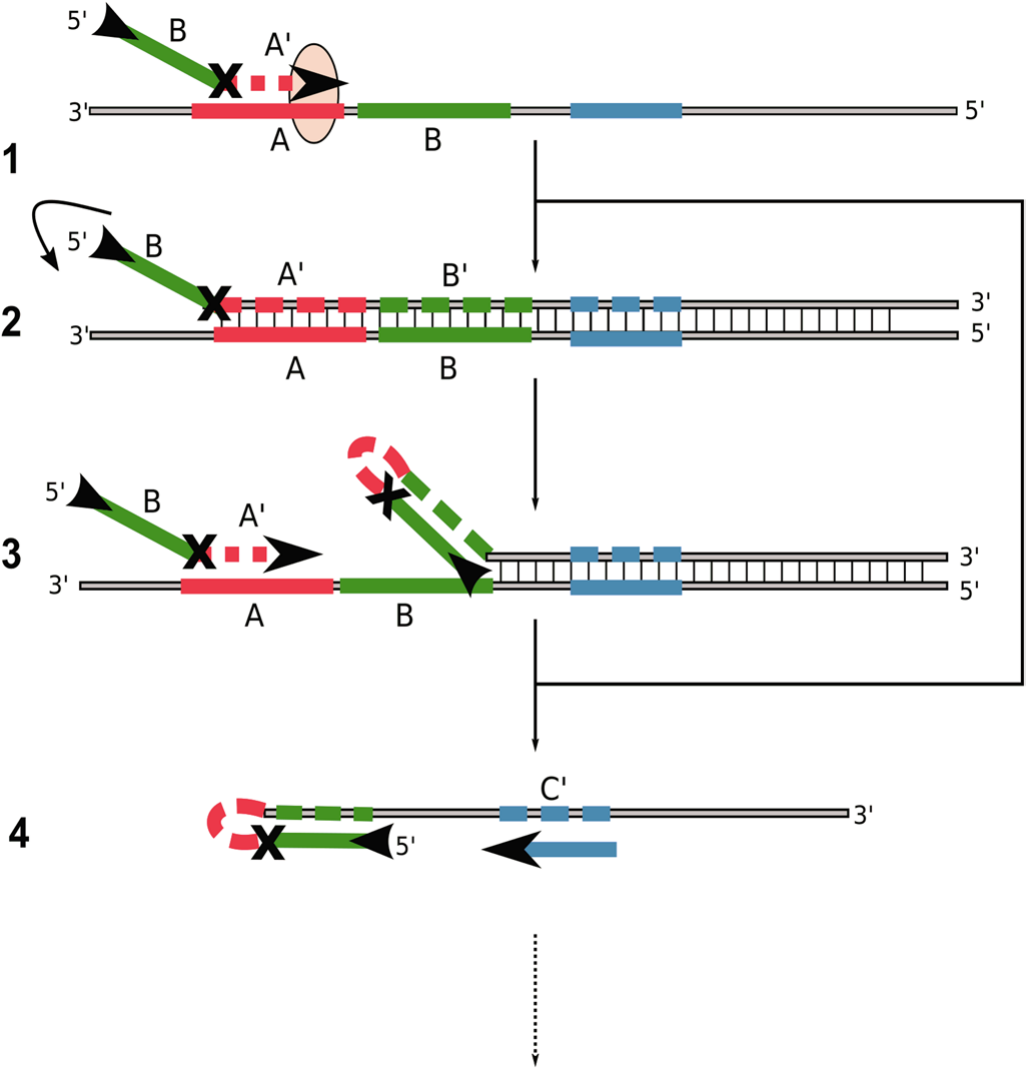
Hinge-initiated primer-based replication with one hinge-primer and PCR-like primer. The hinge-primer anneals to the target region and will be extended to double strand DNA by *Bst* DNA polymerase (1). The refolding to the thermodynamically more stable hairpin structure liberates the initial priming site (2–3). These steps are repeated and the sense DNA strand will be released. The PCR-like primer can anneal to the priming site and will be extended (4).

The hairpin has a higher melting temperature (T_M_) than the primer binding site plus its extension as the double stranded DNA termini alone. This liberates the primer binding site for another annealing/extension step. Therefore, the primer binding-site may have a lower annealing temperature than the secondary binding site in order to promote the hairpin formation. Exemplified with the forward hinge-primer that was used for the PSTVd assay, the primer binding site has a calculated T_M_ of 65 °C and the secondary binding site 68 °C. However, the refolded hairpin end should results in higher T_M_ as the double stranded DNA end without refolding (calculated by mFOLD tool under reaction conditions). Thus, the thermodynamically favourable structure is the hairpin structure which concomitantly results in liberation of another primer binding site. The process can be accelerated by using additional PCR-like outer primer that bind upstream of the hinge-primer or by combining one hinge-primer with a loop-forming primer as known from LAMP. For PSTVd we observed that the best results were achieved by using the hinge-primer in combination with a PCR-like Primer (F3/B3) or a loop-forming primer. The Tp values were between 15 and 30 minutes. We also established that the best linker modification is the abasic site (dSpacer). The hexaethylenglycol spacer (C12-Spacer) resulted in low Tp values in combination with the B3 primer. Other combinations yielded false-positive results, including the control without any linker modification. Especially PSTVd primers comprise strong self-complementary nucleic acid sequences that are susceptible to mispriming. Therefore we designed new primers for the beta-gal region of *Escherichia coli* and included an abasic site as linker modification. To test the performance of the *E*. *coli*-assay we investigated different primer combinations. The lowest Tp values were achieved by using both hinge-primers in one reaction. Additional outer primers did not change the reaction significantly. This indicates that the hinge-primers are highly efficient even in the first amplification steps. To further reduce the complexity of the overall primer design we explored the use of a single hinge-primer either with or without additional corresponding linear primer. The specificity of all reactions could be confirmed by the melting curve analysis which showed distinct peaks at 94 °C. Due to the different signal intensity at the endpoint of each reaction, it can be assumed that the amount of amplified DNA is different between the combinations. The fastest reaction in this set-up could be observed with one reverse hinge-primer including one outer primer. To achieve an exponential reaction, a corresponding linear primer for the reverse direction is required (Fig. 4a, combination 4 and Fig. 7). The simplified reaction mechanism (asymmetric) starts with the annealing of the hinge-primer to the target region and will be extended to double stranded DNA (1). The refolding liberates the initial priming site (2-3). The corresponding linear primer can anneal and will be extended in the reverse direction (4).

The optimal magnesium concentration for most of the assays was determined as 6 mM. Variation of the primer concentration did not affect the reaction significantly. The Tp values were between 37 and 45 minutes in the range of 0.8 to 1.4 µM. When both hinge-primers were used in one common reaction, we always observed false-positive results. Therefore, we propose to use only one hinge-primer in combination with the outer linear primer in an assay. An additional outer primer that binds upstream to the hinge-primer may help to generate single-stranded DNA in the beginning of the reaction akin to outer primers (F3 and B3) used in LAMP^13^. We optimized the amplification speed by decreasing the distance between the priming-site of the hinge-primer and the corresponding outer primer from 160 bp to 77 bp. The decreasing of amplicon size directly correlates with the decrease of the Tp values from 45 minutes to 20 minutes. The correlation also confirms the specificity of the reaction. Finally, we determined the limit-of-detection of our hinge-amplification assay with serially diluted genomic *E*. *coli*-DNA. The lowest reproducibly detectable amount in the linear range was found to be in the range 10^6^ copies.

The results show that the hinge-based amplification assay with a bifunctional primer design generates high molecular weight DNA in an exponential manner. Although, the total amount of amplified DNA was not as high as in LAMP, it can be combined with similar detection methods. In this study, SYBR Green I was used for the detection of the amplified DNA. Yet other DNA-binding dyes or pyrophosphate-based methods that are usually used in LAMP may be evaluated in future experiments. Quantification should be accomplished by real-time monitoring using dsDNA intercalating dyes similar to qPCR. The specific primer sequences can be designed similar to conventional PCR except that higher melting temperatures are required. The hinge primer consists of a priming site as well as a secondary binding site for the refolding sequence. According to the reaction temperature (60–65 °C), the melting temperature can be adjusted by the length of the primer sequences. There is no need to use dedicated software (“PrimerExplorer”) such as in LAMP. Thus, three well chosen binding sites facilitate a high specificity of the assay that is similar to a quantitative PCR assay with two primers and an additional probe for detection^4^. In contrast to LAMP, the lower number of primers in HIP reduces the chance of unspecific priming as consequence of primer interactions, including hetero- and homo-dimers, thus yielding less false-positive results.

As the variation of the primer concentration revealed no decrease of the Tp value, the hinge assay can be performed using lower concentrations (below 1 µM). This is more economic when compared to LAMP that normally requires 1.2 to 1.6 µM of the loop-forming primer to work efficiently. In addition, the risk of mis-priming and the generation of primer-artifacts (dimers) in the absence of a target DNA can be decreased significantly^28, 29^. Although, the investigated sensitivity revealed a linear correlation in the range between 10^6^ to 10^10^ copies, further optimization can lower the detection limit below 10^4^ copies. In contrast to an optimized LAMP assay with a detection limit below 10^3^ copies, the current version of HIP has a higher detection limit and is therefore not applicable for some diagnostic purposes. The lower sensitivity and reaction speed can be attributed to inefficient refolding and priming of the hinge primer. To improve the overall sensitivity of the HIP assay, the length and additional structures of the linker moiety as blocking and refolding element should be investigated more extensively. Since the hinge-initiated primer-based replication is performed under isothermal reaction conditions by the *Bst* DNA polymerase, a PCR-like primer and a modified primer with blocking site, the whole assay can be set up as a low-cost detection reaction. The Bst DNA polymerase is known to tolerate marginal reaction conditions (pH, temperature, salt, inhibitors) and used in portable field assays^30–32^. Thus, highly efficient and economic conditions can be chosen to apply the hinge-amplification to portable field assays comparable to LAMP.

To perform the HIP-assay, we recommend to look for specific primer sequences covering an amplification range of 100 to 150 bp. The hinge-primer consists of a reverse-complementary refolding site and a complementary priming site with 3 to 5 bases distance. Both sites are connected by a short linker such as the dSpacer (abasic site). To achieve exponential amplification, a normal linear primer for the respective reverse direction is required (asymmetric reaction). To accelerate the reaction at the very beginning of the reaction, a third linear primer hybridizing in upstream (5′) to the hinge-primer can be added. This is analogous to the outer primers that are used in LAMP and helps to speed up the initial strand displacement. The required melting temperature should be adjusted to 60 to 65 °C by lowering or increasing the primer length. The reaction itself can be carried out at conditions that are similar to the LAMP-assay. Finally, the direct detection of the amplified double-stranded DNA in the Bst DNA polymerase-based assay can be facilitated by use of intercalating dyes such as ethidium bromide^33^, SYTO-82^34^, SYBR Green I^35^, Evagreen^36^ and berberine^18^. The indirect detection by colour change of calcein^37^ or hydroxynaphthol blue^38^ has to be investigated due to varying amounts of generated pyrophosphate in the HIP compared to the LAMP.

## Conclusion

We describe a new reaction mechanism for isothermal amplification of DNA by using a linker modification (abasic site) that is incorporated between a primer and target binding site of a target-specific oligonucleotide. This linker acts both as a roadblock to Bst DNA polymerase extension as well as a hinge for refolding. The efficient regeneration of the primer binding site and the results in an improved strand-displacement and enables an amplification process to occur under isothermal conditions. It has been demonstrated that a single hinge-primer in combination with a PCR-like primer is sufficient for specific exponential amplification of target DNA. Currently, the new HIP mechanism is not as powerful as LAMP but can be used as basis for further optimization. Yet it may already serve in some settings in which template amounts are less limiting. Our new approach requires no complex primer design, minimal equipment and is as easy to perform as LAMP. Thus, the hinge-initiated primer-dependent amplification (HIP) has the potential to serve as a useful alternative to more complex and cumbersome isothermal amplification techniques also in the field of point-of-care diagnostics.

## Methods

### Generation of sample material

Dried PSTVd-positive material (PV0064) was purchased from the German strain culture collection (DSMZ, Braunschweig, Germany). The RNA extraction was done with the RNeasy Plant Mini Kit (Qiagen, Germany). LAMP-Primers were designed according to Lenarčič *et al*.^39^. The isolated RNA was reverse transcribed into single-stranded DNA using reverse primer and Maxima Reverse Transcriptase (Thermo Scientific, Schwerte, Germany). The DNA template for the LAMP reactions was generated by PCR using the protocol published by Weidemann *et al*.^40^. *E.coli* culture was grown in lysogeny broth (10 g/L tryptone, 5 g/L yeast extract and10 g/L NaCl) at 37 °C with shaking until they reached an optical density at 600 nm of 0.2. The genomic DNA was isolated by using the High Pure PCR Template Preparation Kit (Roche Diagnostics Penzberg, Germany) according to the manufacturer’s instructions. The DNA was quantified by absorption measurements on the Nanodrop ND1000 (NanoDrop Technologies, Wilmington, DE, U.S.A). PCR with the primer LZL-389/LZR-653 was carried out like described by Bej *et al*.^41^. The 360 bp (PSTVd) products were cleaned with the MSB® Spin PCRapace Clean Up Kit (Stratec, Birkenfeld, Germany), quantified by Qubit® dsDNA HS Assay (Life Technologies, Darmstadt, Germany) and used directly in the assays. All oligonucleotides (Thermo Scientific) were HPLC purified.

### LAMP-assay

The PSTVd-LAMP assay was set up in a total volume of 12.5 μL containing 1x supplied Thermopol reaction buffer (New England Biolabs, Frankfurt am Main, Germany), 1.2 mM of each dNTP (Thermo Scientific), 0.8 M betaine (Carl Roth, Karlsruhe, Germany), 0.32 U/µL *Bst* DNA Polymerase (New England Biolabs), 0.8 × SYBR Green I (Thermo Fisher Scientific), 1.6 µM of FIP and BIP primer, 0.4 µM of LF and LR pimer, 0.2 µM of F3 and B3 primer, 6 mM MgSO_4_ (Carl Roth) and purified DNA template.

### Hinge-assay

Hinge-amplification assays were set up in a total volume of 12.5 µL containing 1x Thermo Pol® Reaction buffer, 8 mM magnesium chloride, 0.8 M betaine, 1.0 mM of dNTP-mix, 0.32 U/µL *Bst* 3.0 DNA Polymerase, 0.8x SYBR Green I. Both assays were run in the ESEQuant Tube Scanner (Qiagen) for 60 to 100 minutes at 65 °C while detecting the fluorescence in the FAM channel (Exc.: 470 nm/Em.: 520 nm). According to the experiments, hinge-assays differ in their primer composition as follows: Experiment 1 includes 1.0 µM of PSTVd-ab-HF/R primer. Experiment 2 consists of 1.0 µM of the PSTVd-ab-HF/R primer, PSTVd-HEG-HF/R primer and PSTVd-um-HF/R primer in combination with 1.0 µM of their corresponding forward or reverse primer of the same type, the loop-forming LAMP primer (FIP and BIP) as well as 0.2 µM of the outer LAMP primer (F3 and B3). Experiment 3 was carried out in six positive (with 5 ng genomic *E.coli*-DNA) and negative (without template) reactions. The concentration of the primer LZ-HF and -HR was 1.0 µM, that of the LZL-389 and LZR-653 (outer primer) was 0.2 µM in each reaction. Experiment 4 includes varying concentration of magnesium chloride from 4 to 10 mM. The experiment was carried out in three combinations consisting of 1 µM LZ-HF and LZ-HR, 1 µM LZ-HF with 0.2 µM of outer primer LZL-389 and LZR-653 as well as 1 µM LZ-HR with 0.2 µM of outer primer LZL-389 and LZR-653. Experiment 5 consists of the LZ-HF and -HR primer with concentrations ranging from 0.8 to 1.4 µM. The experiment was carried out in three combinations like described in Experiment 4. Experiment 6 was carried out in four combinations consisting of 1.2 µM of the two hinge-primer (LZ-HF and LZ-HR) with 0.2 µM of the corresponding outer primer LZL-389 and LZR-653, LZF-2 and LZF-2 or LZF-0 and LZR-0. Experiment 7 was performed with varying amount of genomic *E.coli* DNA which was serial diluted 10-fold from 10^10^ down to 10^0^ copies/µL. The copy number was calculated by the equation published by Whelan *et al*.^42^. 1.0 µM of LZ-HR, 0.2 µM of LZF-0 primer and 0.2 µM of the LZR-653 primer were used here. The magnesium concentration was 6 mM.

### Data analysis

To determine the time-to-positivity (Tp) of each reaction, the real-time data were analysed by a mathematical calculation^23^. The analysis of the PSTVd-LAMP and -hinge assays was done by capillary electrophoresis in the Fragment Analyzer (Advanced Analytical Technologies, Inc. Ankeny, USA) according to the instruction of the DNF-915 dsDNA 915 Reagent Kit. The Melting curve analysis was carried out after the amplification in the Light Cycler 480 (Roche) by heating up the samples to 97 °C for 1 minute, cooling down to 55 °C for 2 minutes (4.8 °C/s) and heating up again to 97 °C with 0.05 °C/s while detecting the fluorescence signal continuously.

### Data availability statement

All data generated or analysed during this study are included in this published article (and its Supplementary Information files).

## Electronic supplementary material

Supplementary Information
