# Supplementary material for: Hinge-initiated Primer-dependent Amplification of Nucleic Acids (HIP) – A New Versatile Isothermal Amplification Method
Source: Sci Rep. 2017 Aug 9;7:7683. doi: 10.1038/s41598-017-08067-x (PMC5550438; doi:10.1038/s41598-017-08067-x)
Supplement: Supplementary file 1 — Supplementary Information [file 41598_2017_8067_MOESM1_ESM.pdf]

Supporting Information

"Hinge-initiated Primer-dependent Amplification of Nucleic Acids (HIP) - A New Versatile Isothermal Amplification Method"

Jens Fischbach, Marcus Frohme, Jörn Glökler

1. Results

Table S1 includes the Tp values (time-to-positivity) for the heatmaps. The dataset includes all combinations of the hinge primers (dSpacer, HEG or without modification) with the corresponding reverse primer as either standard PCR primer (F3 and B3) or the loop-forming primer (FIP and BIP) from LAMP. Additional parameters such as R^2 and the signal increase at the time point Tp are used to evaluate the calculated Tp value. Low values indicate no signal increase during amplification or poor quality of the value. The heatmaps in the article (Fig. 3 a-c) represented the HIP reaction with PSTVd-cDNA as template. The negative controls are not included in the heatmap. If samples are determined as false-positive, the corresponding combination is marked with an asterix (*).

Table S1: Analysis of the different primer combinations including dSpacer, HEG or no modification. The Tp values of the positive reactions are used to generate the heatmaps. R^2 describes the quality of the linear fit around the Tp value. The maximal signal increase in the Tp indicates the efficiency of the amplification. HF: Hinge forward primer, HR: hinge reverse primer, ab: dSpacer modification, HEG: C12 modification, um: no modification

Oligonucleotide	Parameter for Tp calculation		
	R^2	Tp [Min.]	signal increase in Tp (dFU/dt) [FU/Min.]
**dSpacer primer**			
PSTVd-ab-HF + B3 pos	1.000	27.25	4.83
PSTVd-ab-HF + B3 neg	0.999	-4.53	0.32
F3 + PSTVd-ab-HR pos	0.998	46.54	1.77
F3 + PSTVd-ab-HR neg	0.912	8.32	0.15
PSTVd-ab-HF + PSTVd-ab-HR pos	0.999	26.89	2.56
PSTVd-ab-HF + PSTVd-ab-HR neg	0.998	0.39	0.43
FIP + PSTVd-ab-HR pos	0.997	38.02	2.05
FIP + PSTVd-ab-HR neg	0.970	-6.30	0.25
PSTVd-ab-HF + BIP pos	0.999	17.78	3.51
PSTVd-ab-HF + BIP neg*	1.000	43.03	3.21
**Hexaethylenglycol primer**			
PSTVd-HEG-HF + B3 pos	0.999	25.25	4.81
PSTVd-HEG-HF + B3 neg	0.428	2.34	0.05

F3 + PSTVd-HEG-HR pos	1.000	52.27	1.43
F3 + PSTVd-HEG-HR neg*	1.000	49.22	4.44
PSTVd-HEG-HF + PSTVd-HEG-HR pos	0.999	45.68	3.06
PSTVd-HEG-HF + PSTVd-HEG-HR neg	0.938	-6.75	0.22
FIP + PSTVd-HEG-HR pos	1.000	45.67	3.04
FIP + PSTVd-HEG-HR neg*	1.000	47.18	0.3
PSTVd-HEG-HF + BIP pos	0.999	47.54	2.53
PSTVd-HEG-HF + BIP neg*	1.000	53.10	3.53
**Unmodified primer**			
PSTVd-um-HF + B3 pos	0.961	0.65	0.31
PSTVd-um-HF + B3 neg	0.952	-5.04	0.18
F3 + PSTVd-um-HR pos	1.000	48.56	4.59
F3 + PSTVd-um-HR neg	0.962	-2.15	0.53
PSTVd-um-HF + PSTVd-um-HR pos	0.997	44.57	4.68
PSTVd-um-HF + PSTVd-um-HR neg*	1.000	49.92	4.87
PSTVd-um-HF + BIP pos	0.996	42.06	7.36
PSTVd-um-HF + BIP neg	0.980	-2.76	0.34
FIP + PSTVd-um-HR pos	0.984	0.80	2.31
FIP + PSTVd-um-HR neg*	1.000	49.98	5.62
**LAMP primer**			
FIP + B3 pos	1.000	43.07	3.38
FIP + B3 neg	0.978	-8.44	0.14
F3 + BIP pos	1.000	52.99	2.95
F3 + BIP neg	1.000	-4.75	0.17
FIP + BIP pos	1.000	46.31	2.88
FIP + BIP neg	1.000	-4.84	0.17
F3 + B3 pos	1.000	-10.38	0.12
F3 + B3 neg	0.246	-21.27	0.04
